# Deployment of attractive targeted sugar baits in western Zambia: installation, monitoring, removal, and disposal procedures during a Phase III cluster randomized controlled trial

**DOI:** 10.1186/s12936-024-05030-w

**Published:** 2024-07-09

**Authors:** Erica Orange, Irene Kyomuhangi, Mundia Masuzyo, Mwansa Mwenya, Patricia Mambo, Kochelani Saili, Chama Chishya, Javan Chanda, Ruth A. Ashton, Thomas P. Eisele, Joshua Yukich, John Miller, Kafula Silumbe, Busiku Hamainza, Joseph Wagman, Annie Arnzen, Angela F. Harris, Julian Entwistle, Laurence Slutsker, Thomas R. Burkot, Megan Littrell

**Affiliations:** 1 PATH, Seattle, WA USA; 2 Centre for Applied Malaria Research and Evaluation, Tulane School of Public Health and Tropical Medicine, New Orleans, LA USA; 3 Present Address: Centre for Health Informatics Computing and Statistics, Lancaster University, Lancaster, UK; 4 PATH, Kaoma, Zambia; 5 Present Address: Macha Research Trust, Choma, Zambia; 6 PATH, Lusaka, Zambia; 7 National Malaria Elimination Centre, Lusaka, Zambia; 8 PATH, Washington, DC USA; 9 Innovative Vector Control Consortium, Liverpool, UK; 10 Independent Consultant, Atlanta, GA USA; 11 Australian Institute of Tropical Health and Medicine, James Cook University, Cairns, Qld Australia

**Keywords:** Malaria, Vector control, Attractive targeted sugar bait

## Abstract

**Background:**

Attractive Targeted Sugar Baits (ATSBs) offer a complementary vector control strategy to interventions targeting blood feeding or larval control by attacking the sugar feeding behaviour of adult mosquitoes using an attract-and-kill approach. Western Zambia was the first location to receive and deploy ATSB Sarabi version 1.2 stations in a Phase III cluster randomized controlled trial. This paper describes ATSB station installation, monitoring, removal, and disposal, quantifies ATSB station coverage, and reports major reasons for ATSB station replacement.

**Methods:**

ATSB stations were deployed during two annual transmission seasons, through scheduled installation and removal campaigns. During deployment, monitoring was conducted per protocol to maintain high coverage of the ATSB stations in good condition. Routine monitoring visits during the trial captured details on ATSB station damage necessitating replacement following pre-defined replacement criteria. Annual cross-sectional household surveys measured ATSB station coverage during peak malaria transmission.

**Results:**

A total of 67,945 ATSB stations were installed in Year 1 (41,695 initially installed+ 26,250 installed during monitoring) and 69,494 ATSB stations were installed in Year 2 (41,982 initially installed+ 27,512 installed during monitoring) across 35 intervention clusters to maintain high coverage of two ATSB stations in good condition per eligible household structure. The primary reasons for ATSB station replacement due to damage were holes/tears and presence of mold. Cross-sectional household surveys documented high coverage of ATSB stations across Year 1 and Year 2 with 93.1% of eligible structures having ≥ 2 ATSB stations in any condition.

**Discussion:**

ATSB station deployment and monitoring efforts were conducted in the context of a controlled cRCT to assess potential product efficacy. Damage to ATSB stations during deployment required replacement of a subset of stations. High coverage of eligible structures was maintained over the two-year study despite replacement requirements. Additional research is needed to better understand the impact of damage on ATSB station effectiveness under programmatic conditions, including thresholds of threats to physical integrity and biological deterioration on product efficacy.

**Conclusions:**

Optimizing ATSB stations to address causes of damage and conducting implementation research to inform optimal delivery and cost-effective deployment will be important to facilitate scale-up of ATSB interventions.

## Background

The scale-up of successful malaria interventions, including insecticide-treated nets (ITNs) and indoor residual spraying (IRS), has averted nearly two billion malaria cases and 12 million malaria deaths over the past two decades [1, 2]. While ITNs and IRS have been the pillars of malaria vector control and transmission reduction for the past two decades, their continued effectiveness is threatened due to evolving mosquito physiologic and behavioural resistance including daytime and outdoor biting and resting characteristics [1–3]. The current vector control toolbox requires new paradigms to mitigate malaria transmission that can complement ITNs and IRS in an integrated vector control approach [4].

In addition to the biological need for female *Anopheles* mosquitoes to take a blood meal for egg production, they also must regularly feed on sugar to survive [5]. Attractive Targeted Sugar Baits (ATSBs) offer a complementary vector control strategy by employing the attract-and-kill approach to exploit the sugar feeding behaviour of adult mosquitoes [6, 7]. Most ATSBs are designed specifically to attract mosquitoes to a source of sugar inducing them to ingest a toxicant that kills them. By employing this novel approach, ATSBs have the potential to combat the challenges of resistance to the contact insecticides used in ITN and IRS and, in addition, can be deployed outdoors [6, 7].

Since 1965, significant progress has been made in the use of sugar baiting for mosquito and malaria vector control—ranging from spraying toxic bait directly onto vegetation, homemade apparatuses with fermented fruit, and the deployment of mounted bait stations [7–10]. Attractants used to lure mosquitoes to feed on sugar baits have included engineered aromatic compounds, fruit-based sources, and plant-based sources [7]. Toxicants used in ATSBs include synthetic chemical insecticides, biopesticides, and plant-based products [8]. Attractants that can effectively lure mosquitoes, coupled with an active ingredient that kills them upon ingestion and a sustainable deployment mechanism, are key to the success of ATSBs as a new vector control paradigm.

From 2014 to 2018, laboratory, semi-field, and field studies in Mali and Israel of prototype Sarabi ATSB devices from Westham Ltd. (Hod-Hasharon, Israel) demonstrated that large proportions of target *Anopheles* populations fed from mounted bait stations, and female *Anopheles gambiae *sensu lato* (s.l*.) density was reduced in villages receiving ATSBs [11]. Proof-of-concept studies in Mali in 2016 and 2017 and in Zambia in 2021, with prototype Sarabi ASB devices (attractive sugar bait devices without toxicant), found *Anopheles funestus* and *An. gambiae* vector populations readily fed from ASB devices. The proof-of-concept studies also found that the observed ASB feeding rate, according to modelling, was in line with feeding sufficient to reduce malaria incidence by at least a further 30% when two ATSB stations were installed on exterior walls of household structures and used in combination with standard-of-care vector control (IRS or ITN) [12, 13].

To generate evidence of public health impact required by the World Health Organization (WHO) to open a new malaria vector control product class for ATSBs, as well as contribute to the prequalification of the Sarabi ATSB station product, Phase III trials with epidemiological and entomological endpoints were conducted in Mali, Kenya, and Zambia between 2021 and 2024 [14]. The trial site in western Zambia was the first location to receive and deploy ATSB stations in large quantities as part of the Phase III trials. This paper describes ATSB station deployment including installation, monitoring, removal and disposal; quantification of ATSB station use and coverage; and assessment of ATSB station conditions requiring replacement, as implemented in the Zambia trial setting.

## Methods

A two-arm, cluster-randomized controlled trial (cRCT) was conducted in Western Province, Zambia to determine the impact of ATSB stations in the context of universal vector control (ITNs and/or IRS), compared to universal vector control alone (the standard of care) on clinical malaria incidence [14]. The cRCT in Zambia was implemented in 70 study clusters (35 intervention clusters and 35 control clusters) across Kaoma, Luampa, and Nkeyema districts, representing a population of 122,023 people in 23,466 households across all 70 clusters at the start of the trial (based on pre-trial enumeration estimates). Trial clusters were drawn with a minimum of 250 households per cluster to meet sample size requirements [14]. The trial measured epidemiological and entomological outcomes during a two-year seasonal transmission deployment of ATSB stations in intervention clusters (Year 1—November 2021–June 2022; Year 2—November 2022–June 2023) (Table 1). The trial design incorporated standards for implementation and monitoring of the ATSB station intervention to establish efficacy. Full details of the study site are available in Arnzen et al*.* [15].

**Table 1:**
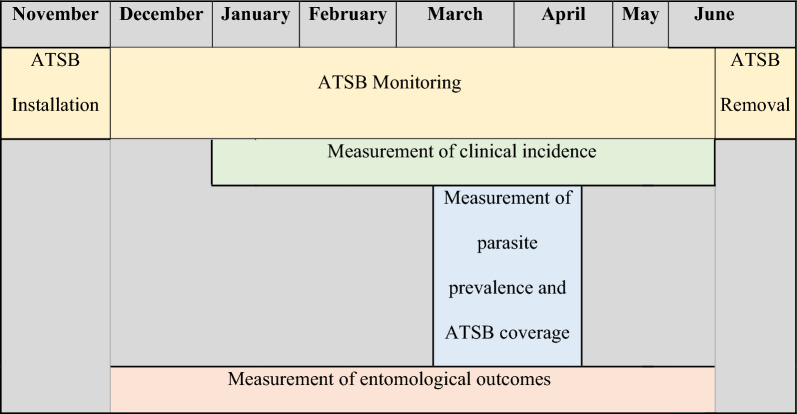
Timeline of ATSB Seasonal deployment in relation to trial endpoint measurements, Years 1 and 2

### ATSB station product

The ATSB station in the cRCT was the ATSB Sarabi v1.2 bait station, manufactured by Westham Co. (Hod-Hasharon, Israel). These stations contained a bait made from date syrup mixed with dinotefuran as the active ingredient. Dinotefuran (N-methyl-N′-nitro-Nʺ-[(tetrahydro-3-furanyl)methyl]guanidine) (MITSUI CHEMICALS, Inc.) is a neonicotinoid insecticide that rapidly kills mosquitoes [16]. Additionally, the ATSB contained a bittering agent, Bitrex^®^ (Johnson Matthey), to deter human consumption of the bait. Environmental assessments prior to the cRCT suggested that the toxicant posed limited risk to non-target organism and the environment when deployed within the bait station reservoir and retrieved for proper disposal (ERM, 2021, A. Harris, personal communication).

**Fig. 1:**
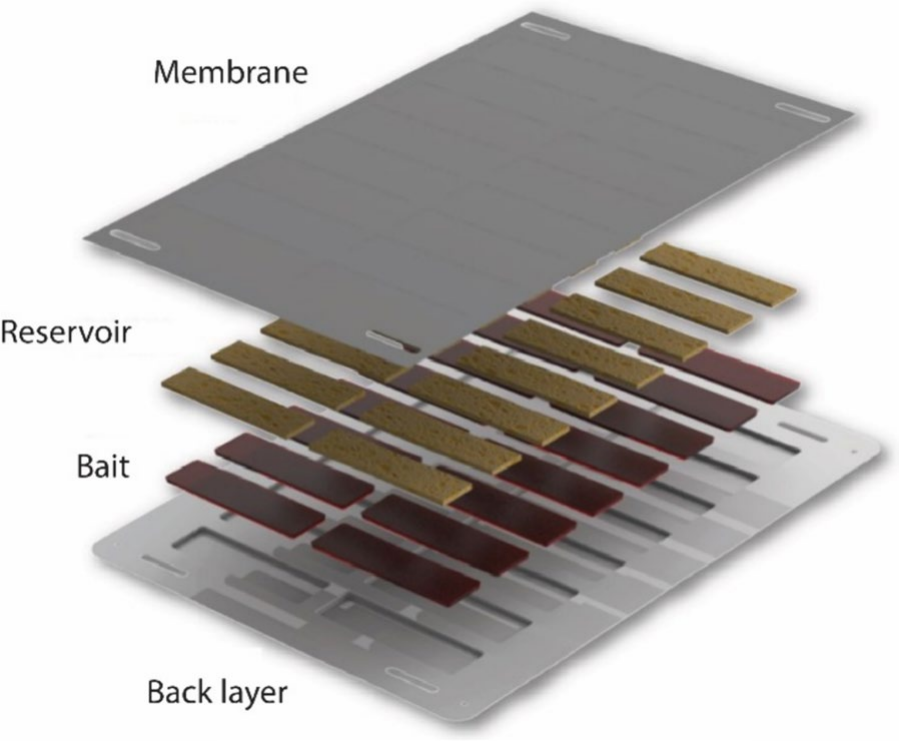
ATSB Sarabi v1.2 design

The bait was contained within the bait station in 16-cell sponge-like reservoirs. The bait and reservoirs were sealed by a black membrane to a plastic back layer. The black membrane had tiny perforations allowing mosquitoes to feed on the bait while preventing insects without sucking mouthparts from feeding from it. The plastic back layer was equipped with large holes in each corner for ease of installation (Fig. 1).

Each ATSB station deployed throughout the study was marked with a unique pre-printed QR code to allow tracking over time and association with specific batch manufacturing information.

### ATSB station storage and handling

Packaged ATSB stations were transported by air and sea to Zambia and then within Zambia by flatbed semitruck to the study site (Kaoma, Western Province). Following the manufacturer’s guidance, ATSB stations in their sealed, protective packaging, were stored in Kaoma in a well-ventilated storage facility, free from water, fire, direct sunlight, and rodents. The storage facility and transportation mechanisms maintained the recommended storage temperature (between 0 and 60 degrees Celcius) confirmed by temperature data loggers.

ATSB stations were transported to each intervention cluster by sport utility vehicle and distributed to community-based members trained as ATSB monitors for installation and replacement. ATSB stations remained in their original packaging, inclusive of cardboard boxes with silver preservation bags and bubble wrap, until installed on eligible structures. ATSB monitors handling the ATSB stations wore disposable latex gloves for proper handling and removal of ATSB stations. No wiping or cleaning of the ATSB stations was conducted by ATSB monitors during any study phase.

### ATSB station installation

ATSB stations were installed on eligible structures in intervention clusters through mass installation campaigns. Campaigns were conducted prior to each annual transmission season, between 1 and 13 November 2021, and 31 October–12 November 2022, with all ATSB stations installed two weeks prior to the enrolment of the epidemiological cohort and start of entomological surveillance. Installation teams were comprised of trained and supervised community members who worked together to sensitize and consent households, install ATSB stations, and conduct data entry on each installed ATSB station. Two hundred and eighty individuals were recruited and supervised during each two-week installation campaign to install ATSB stations across the 35 intervention clusters. Additional information on community sensitization prior to ATSB station installation is available elsewhere [17].

**Fig. 2:**
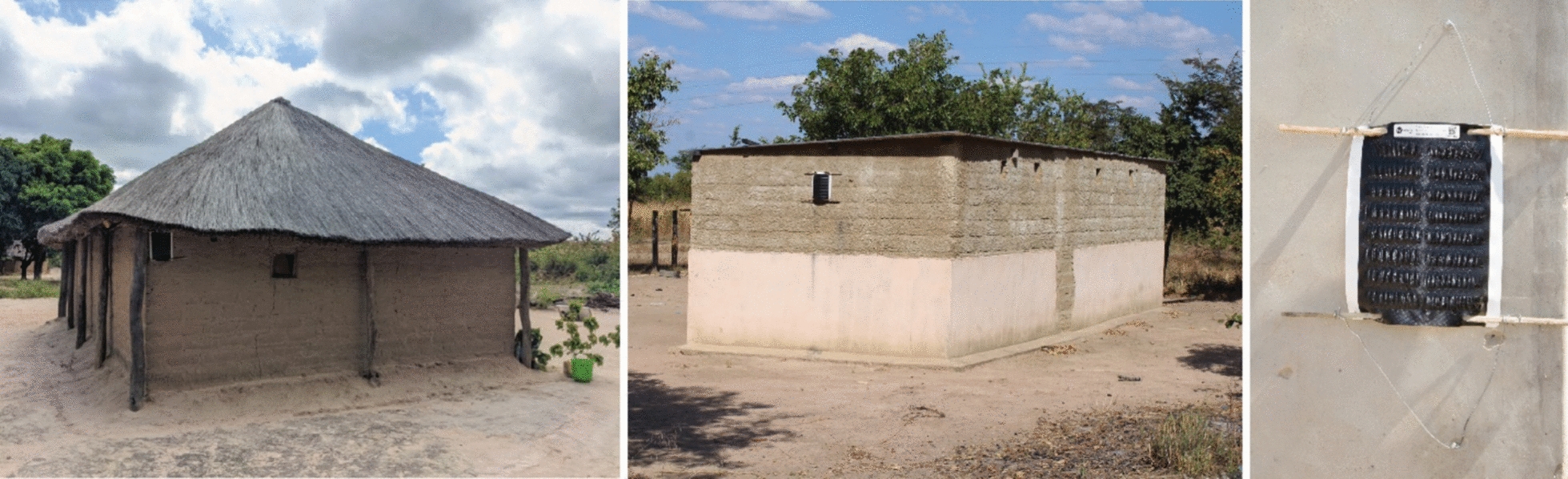
ATSB installation on eligible structures

Heads of households provided consent prior to ATSB station installation. At each eligible structure within the household, two ATSB stations were hung using a combination of bamboo sticks, wires, string, and nails to attach them to the exterior wall, depending on the most suitable material for the wall structure composition. The ATSB stations were installed in protected locations, where possible, such as close to the roof overhang or under an eave and a minimum of one meter above the ground (Fig. 2). ATSB stations were installed on opposite walls of the structure unless adjacent walls offered better protection from rain, sun, and wind.

Structures eligible for ATSB station installation were defined as residential structures with a complete roof, at least three complete walls, and walls at least one meter in height. Households within the trial site had an average of 1.6–2.1 eligible structures, receiving two ATSBs per eligible structure. This primarily resulted in installation on sleeping structures and multi-use residential structures. For apartment-style flats with exterior walls exceeding 10 m in length, two ATSB stations were installed every 10 m or per residential apartment. Non-residential buildings (e.g., shops, schools, churches, tobacco sheds, animal kraals, toilets, bathing shelters, and food storage shelters) were not eligible for ATSB station installation due to product integrity protection and cultural considerations. At the time of installation, the location and the QR code of installed ATSB stations were captured using a digital data collection tool (CommCare, Dimagi, Cambridge MA) on android mobile devices.

### ATSB station monitoring

Two to four community-based ATSB monitors per cluster (a total of 79 from November 2021-June 2022 and 87 from November 2022-June 2023), were recruited, trained, and supervised to conduct ATSB monitoring visits. The aim of ATSB monitoring visits was to maintain ongoing, high coverage of ATSB stations in good condition, defined as two ATSB stations per eligible structure that did not meet the replacement criteria for damage. ATSB monitoring included installation of new ATSB stations on newly constructed eligible structures and on eligible structures where ATSB stations were missing; replacement of damaged ATSB stations per pre-defined replacement criteria (Table 2); removal of excess ATSB stations that may have been relocated to the structure (e.g., > 2 ATSB stations); and removal of discarded ATSB stations.

**Table 2:** Pre-defined replacement criteria for damaged ATSB stations

Damage to the ATSB	Criteria for replacement	Further guidance
Hole, tear, or puncture	1 or more cell is completely torn open	Completely torn open means that the inside of the bait station and/or white plastic is fully visible
Leaking: bait/liquid is coming off the black membrane onto the nearby surroundings	On to white border or off the ATSB station (onto wall or ground)	Leaking does not include “sweating” or “sticky” surfaces. Sweating means that the ATSB station has absorbed some water, from recent rains, bringing liquid to the surface but the liquid is not leaking off the bait station
Mold: fuzzy growth on the surface of the membrane	Spots of mold that are larger than the rubber end of a pencil OR A layer of black mold spreading across more than half the ATSB station	Mold often looks like white, black, reddish or brown fuzz. Spots of mold larger than the rubber end of a pencil may include spots of mold that are ‘touching’
Bait is depleted (flat, empty cells)	8 or more cells without liquid	Depletion is determined by using a gloved hand to pinch the cell and confirm if it is empty between the membrane and the plastic Depletion cannot be determined from looking alone, as in some circumstances the black membrane may look ‘stretched out’ from water retention, though it is not depleted of bait
Dirty/mud	8 or more cells completely covered in dirt	Completely covered cells means that dirt, mud, paint or thick dust could not easily come off

**Fig. 3:**
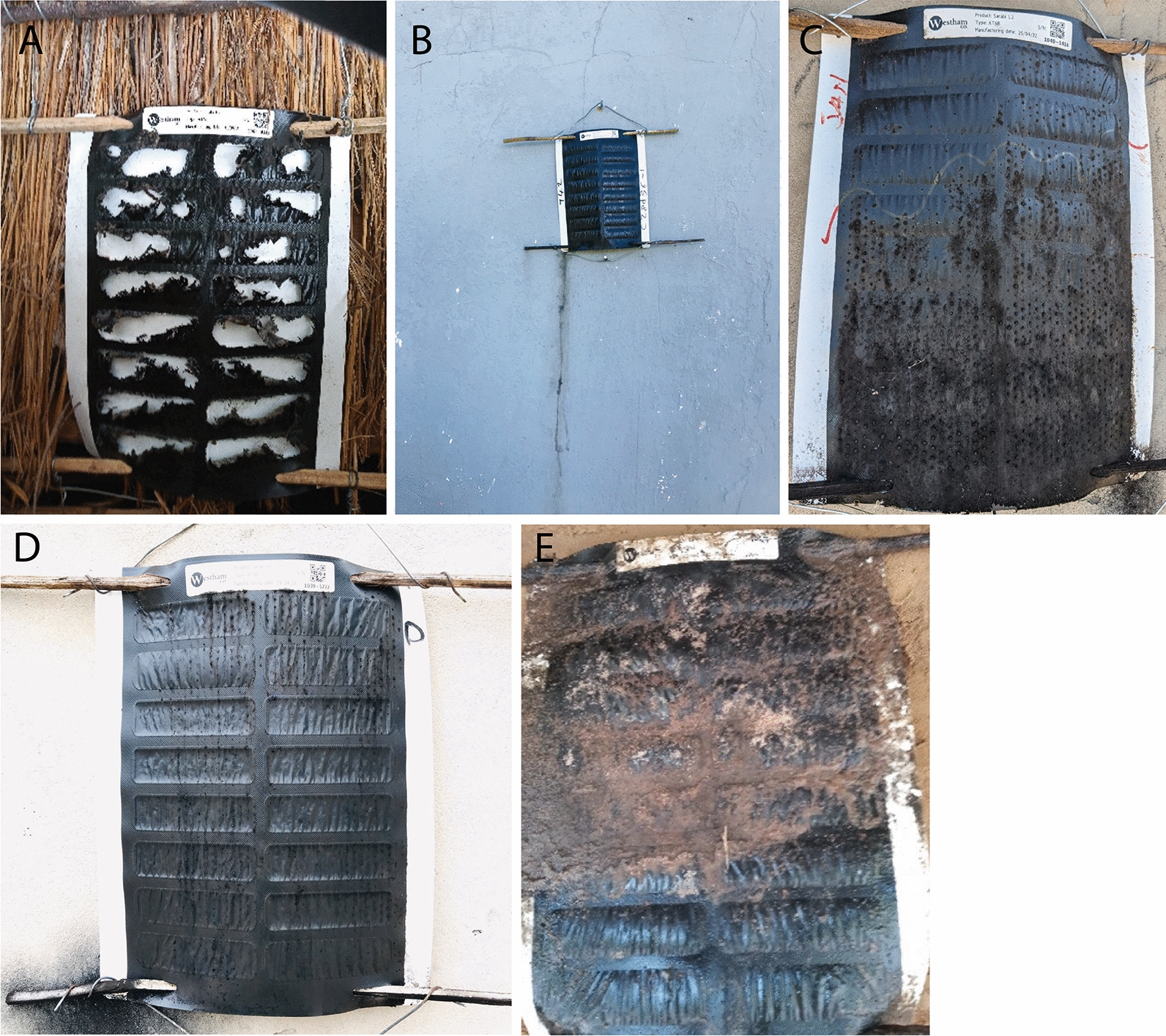
ATSB damage types. ATSB Damage Types **A** Holes/tears caused by rodents; **B** Leaking onto wall; C* Spots of mold that are larger than the rubber end of a pencil and a layer of black mold spreading across more than half the ATSB station; D* Depletion of more than 8 cells; **E** Dirt covering more than 8 cells. *C, **D** also include mold growth from the bamboo stick onto the structure wall. Mold growth from the bamboo stick onto the structure was not a criterion for replacement

Routine monitoring visits were made to each installed ATSB station at least once every two months during the deployment period (December-June). During each visit, ATSB monitors used a digital data collection tool (CommCare, Dimagi, Cambridge MA) to assess and record the condition of each ATSB station. Based on the assessment, ATSB monitors were instructed by CommCare to remove ATSB stations meeting any of the pre-defined replacement criteria (Table 2) and replace it with a new ATSB station. Between routine monitoring visits, ATSB monitors also responded to reports of ATSB station damage from households. ATSB stations were eligible for replacement as soon as they met the replacement criteria for damage and therefore could be replaced multiple times in a monitoring round. Each visit to an ATSB station, either for routine monitoring or responding to damage, was considered an ATSB monitoring visit. Data on the date of replacement, location, photo, QR code of the removed ATSB station, and QR code of the new ATSB station were captured. Examples of ATSB stations eligible for replacement are shown in Fig. 3.

The ATSB replacement criteria for damaged ATSB stations were agreed together with teams involved in the equivalent trials in Mali and Kenya prior to the start of the Zambia trial. Replacement criteria for damaged ATSB stations were chosen to try to ensure product integrity to avoid potential loss of efficacy, to maintain product attractancy to target vectors, for community acceptance, and for protection of human and environmental health. Due to the speed for which minor mold growth was appearing, in February 2022, the replacement criteria for mold were revised to allow for some initial mold growth before reaching a level that would require replacement. Guidance for replacement due to mold was changed from mold affecting “any of the cells and larger than the end of a pencil OR 5 or more cells (any size mold)” to “spots of mold that are larger than the rubber end of a pencil OR black mold spreading across half of the ATSB.” At the same time, guidance for replacement due to holes/tears was revised for the remainder of the trial from holes/tears affecting “any of the cells” to “one or more cell is completely torn open” to provide further clarity on hole assessment. In March 2023, replacement due to holes from rodent damage was restricted to once per week due to a high volume of replacements for this reason.

In addition to assessing the condition of the ATSB station, intervention coverage was monitored at each household by checking each eligible structure, including newly built structures, to confirm the presence of two ATSB stations. If an eligible structure was missing one or more ATSB stations, a new ATSB station was installed. If an eligible structure had more than two ATSB stations installed (e.g., from being relocated from a nearby structure), the additional ATSB station was removed. Lastly, ATSB monitors captured details on any discarded ATSB stations found during monitoring visits and properly disposed of them.

In the 1st year of the ATSB deployment (November 2021–June 2022), data collected during an ATSB monitoring visit guided monitors to indicate whether or not the ATSB station met criteria for replacement up to first replacement criterion met (beginning with holes/tears and followed in order by leaks, mold, depletion, and dirt). After one replacement criterion was met, the form skipped to the end of the assessment and guided the monitor to replace the ATSB station. In the 2nd year of ATSB deployment (November 2022–June 2023), the ATSB monitoring tool guided monitors to assess whether an ATSB station met each criterion for replacement for a more comprehensive assessment of the ATSB station condition.

### ATSB station removal and disposal

ATSB stations were removed from all structures through scheduled mass removal campaigns at the end of each annual transmission season, after all epidemiological cohort visits and entomological measurements had concluded. Removal campaigns were conducted between 15 and 30 June 2022 and 15–30 June 2023. Approximately 230 individuals were recruited and supervised during each two-week removal campaign across the 35 intervention clusters each year. Teams of trained community members worked together during the removal campaigns to sensitize households, collect data on the location and condition of each station, and remove and dispose of them.

During the 2nd year of ATSB station removal, additional details on the structure wall composition, structure roof composition, ATSB station placement position on the structure, and level of ATSB station protection provided by an eave or roof overhang were captured at the time of removal.

Removed ATSB stations and other potentially contaminated supplies, such as used gloves and bubble wrap from packaging, were placed in large biohazard bags. Full biohazard bags were collected from each cluster throughout ATSB installation, monitoring and removal campaigns, and taken to Kaoma for sealing in 210 L plastic drums. ATSB drums were transported by flatbed semitruck to a facility in Lusaka, where they were incinerated at > 1000 degrees Celsius, in accordance with manufacturer’s guidance. All other non-contaminated materials, such as cardboard boxes from shipment, were disposed of locally.

### ATSB station coverage

ATSB station coverage was measured through an annual cross-sectional household survey conducted in March–April 2022 and 2023, concurrent with the peak malaria transmission season and ATSB station monitoring. Participating households were selected in each cluster each year by simple random sample from a sampling frame of enumerated households. Each household survey included an inspection of household structures for ATSB stations to assess their presence and condition, together with the collection of other household-level indicators. ATSB station coverage was defined as ‘high’ coverage if ≥ 90% of eligible structures had ≥ 2 ATSB stations in any condition. Household survey procedures are described in detail elsewhere (Ashton et al. in preparation).

### ATSB data management

ATSB stations were tracked at each visit (e.g., installation, monitoring visits, and removal) by the unique QR code and location coordinates. ATSB station data were downloaded routinely from CommCare and analysed to inform monitoring activities. Photo reviews were conducted to validate ATSB station monitoring quality and types of ATSB station damage. Two ATSB supervisors routinely used data to prioritize supervision of ATSB monitors and perform quality assurance spot checks.

Analyses of routine monitoring data were conducted in the R programing language (R Foundation for Statistical Computing, Vienna, Austria), indicators were tracked in MS Excel (Microsoft, Bellevue WA), and spatial monitoring conducted using QGIS (Open Source Geospatial Foundation Project) and the shiny web application (R package version 1.8.0.9000). Outputs included metrics on ATSB station installations, ATSB station monitoring visits, ATSB station damage and replacement, and ATSB station removal, as well as maps enabling the geographic visualization of these metrics to confirm where ATSB station monitoring visits occurred.

A subset of ATSB stations were removed in Year 1 as part of a bio-efficacy study [18] and in Year 2 as part of an ATSB durability study (Karabo et al. in preparation). ATSB stations included in these studies were monitored through separate complimentary datasets.

## Results

A total of 41,695 ATSB stations were installed in November 2021 and 41,982 ATSB stations installed in November 2022 across the 35 intervention clusters during the installation campaigns. The difference between installation campaign totals between Year 1 and Year 2 can be attributed to an increase in acceptance of the intervention at the time of installation and to additional eligible structures built during the trial period. As shown in Table 3, during Year 1 of ATSB monitoring visits (December 2021–June 2022), 26,250 new ATSB stations were installed to replace ATSB stations meeting the damage criteria or due to absence of an ATSB station on an eligible structure. Similarly in Year 2 of ATSB monitoring (December 2022–June 2023), 27,512 new ATSB stations were installed during monitoring visits. By the end of each deployment year, approximately two-thirds of the quantity of ATSB stations required during the installation campaigns were needed across the intervention arm to maintain high coverage of ATSB stations in good condition (63% in Year 1—26,250/41,695; and 66% in Year 2—27,512/41,982).

**Table 3:** ATSB station installation summary

	Year 1 (Nov 2021–Jun 2022)	Year 2 (Nov 2022–Jun 2023)
# of ATSB stations installed during initial installation campaign (*November*)	41,695	41,982
# of ATSB stations installed during monitoring as replacements for damaged ATSB stations and to fill gaps in coverage	26,250	27,512
Total # of ATSB stations installed	67,945	69,494

In Year 1 and Year 2, the majority of ATSB stations were replaced in February–March, three to four months following the installation campaigns. Replacements during Year 1 were not directly comparable to Year 2 due to the changes in the replacement criteria for mold and holes/tears in February 2022 and the addition of data collected on multiple damage criteria in Year 2 (Table 4).

**Table 4:** ATSB monitoring visit and replacement summary

	Year 1—# of ATSB monitoring visits n	Year 1 ATSB monitoring visits where ATSB station met damage criteria n (%)	Year 2—# of ATSB monitoring visits n	Year 2 ATSB monitoring visits where ATSB station met damage criteria n (%)
Nov*	984	103 (10.5%)	N/a	N/a
Dec-Jan​	41,999	5,138 (12.2%)	48,719	4341 (8.9%)
Feb-Mar​	39,830	7,478 (18.8%)	49,122	9103 (18.5%)
Apr-May	41,029	4,956 (12.1%)	50,549	7202 (14.2%)
Jun 1–10**	16,501	838 (5.1%)	1931	1349 (69.9%)
**Total**	140,343	18,513 (13.2%)	150,321	21,995 (14.6%)

^*^ Monitoring visits conducted in November 2021 were to respond to reported ATSB removal in communities. New ATSB stations were installed following additional community sensitization efforts

^**^ Monitoring visits continued into early June of Year 1 and Year 2 to maintain high coverage through the end of the epidemiological cohort study and entomological collections. In Year 2, an emphasis was placed on responding to damaged ATSB stations reported by households

The primary reasons for ATSB station replacement due to damage were holes/tears and mold, as shown in Table 5. Holes/tears were frequently due to rodents feeding on one or more cells of the ATSB station, resulting in cell(s) meeting the criterion of being completely torn open. Holes/tears were the primary reason for replacement each month from December through June, except February when mold was the primary reason for replacement. Mold growth was commonly observed throughout the study period, with most mold growth reaching replacement eligibility in February and March. No severe adverse events were reported in relation to the ATSB product or ATSB damage as part of the trial.

**Table 5:** Number of damaged ATSB stations by damage type, including damage identified in bio-efficacy and durability monitoring datasets

# of damaged ATSB stations due to	Year 1 (Nov 2021–Jun 2022)	Year 2 (Nov 2022–Jun 2023)
Holes/tears	7,637	12,670
Leaks	2,426	959
Mold	7,085	6009
Depletion	877	553
Dirt	495	353
Multiple factors*	n/a	1870

* In Year 1, data captured up to first reason for replacement due to damage in the order appearing in the table, thus multiple factors were not assessed. In Year 2, all reasons for replacement were captured. In cases of multiple criteria for replacement, this is reported as multiple factors. This table should be interpreted as ‘holes first’ for Year 1 and ‘holes only’ for Year 2

A total of 38,196 ATSB stations were removed in June 2022 and 41,096 ATSB stations removed in June 2023 across the 35 intervention clusters during the scheduled removal campaigns (Table 6). After the conclusion of monitoring activities and the end of the removal campaign, there was a small number of ATSB stations unaccounted for in both years. ATSB stations were unaccounted for primarily due to unintentional removal from adverse weather or intentional removal by household members or members of the community. In Year 1, intentional removal of the ATSB stations by community members was more prevalent in the earlier part of the year when ATSB stations were first deployed, necessitating targeted community engagement to further introduce the ATSB station product.

**Table 6:** ATSB station removal summary

	Year 1 (Jun 2022)	Year 2 (Jun 2023)
#of ATSB stations removed during removal campaign (*June*)	38,196	41,096
#of ATSB stations that were eligible for replacement due to damage at the time of scheduled removal	5306	5086
%of ATSB stations that were eligible for replacement due to damage at the time of scheduled removal	13.9%	12.4%

Data from the cross-sectional household surveys show that 93.1% of eligible structures had ≥ 2 ATSB stations in any condition, demonstrating a high coverage of ATSB stations achieved across Year 1 and Year 2 (Table 7). In comparison, only 71.5% of eligible structures had ≥ 2 ATSB stations not meeting the replacement criteria, demonstrating the extent of ATSB stations affected by damage throughout the trial.

**Table 7:** ATSB station coverage

	Year 1 (Mar-Apr 2022)	Year 2 (Mar-Apr 2023)	Y1 and Y2 combined
	n = 981	n = 1,400	n = 2,381
Among eligible structures assessed, % (95% CI) with ≥ 2 ATSB stations in any condition	98.3% (97.5–99.0)	89.5% (87.1–91.9)	93.1% (91.6–94.7)
Among eligible structures assessed, % (95% CI) with ≥ 2 ATSB stations in good condition	78.0% (72.8–83.4)	66.9% (61.4–72.3)	71.5% (67.1–75.8)

## Discussion

Systematic, standardized deployment and intensive monitoring efforts resulted in high ATSB station coverage throughout two seasonal deployments of ATSB stations for a Phase III cRCT efficacy trial. High ATSB station coverage was also due in part to intensive community engagement efforts which included targeted responses to reports of damaged, missing, or removed ATSB stations. Despite routine monitoring and replacement according to protocol, gaps in coverage of ATSB stations in good condition (i.e., not meeting criteria for replacement due to damage) were observed. Data from ATSB station monitoring suggest that approximately 14% of ATSB stations met replacement criteria due to damage at any given time. The deployment and monitoring methods used in the trial suggest the need for continuous replacement strategies as part of ATSB intervention deployment, and suggest a need for an improved understanding of the impact of damage on ATSB station efficacy.

The main threats to ATSB station physical integrity observed during deployment in western Zambia were the development of holes/tears on the bait station membrane and mold growth. Although it is known that ATSB stations in good condition remain efficacious throughout a seasonal deployment in this context [18], it is not known if threats to physical integrity, including holes/tears and presence of mold, may be associated with reduced product efficacy (e.g., due to reductions in product attractancy and vector feeding). Cage and semi-field studies using the Sarabi ATSB v1.2 in Kenya suggest that mold may not be a major deterrent to *Anopheles* mosquito attractancy and feeding on the bait station [19]. Further research may be warranted to identify with greater specificity the thresholds with which mold and holes/tears are associated with reduced efficacy in a field setting. If demonstrated to maintain efficacy, relaxed replacement criteria may reduce replacement and resource needs for scaling up the intervention.

It is important to consider that ATSB station damage observed in western Zambia is likely influenced by contextual factors in the region, such as the relationship between rain, levels of structure protection [15], and the subsequent development of leaks and mold. Other contextual considerations include factors that may contribute to frequency of holes/tears affecting the bait station membrane, such as local rodent species, location of ATSB station installation for easy access by local rodent species, food storage and farming practices, and housing materials. Additional research on ATSB station deployment in other settings is needed to better understand ATSB damage and threats to physical integrity across different contexts.

Commonly observed ATSB station damage may negatively impact ATSB acceptability among community members. A qualitative study conducted in the ATSB trial area found that mold and leaks were associated with negative perceptions of the ATSB station due to associated discoloration and destruction of structure walls. However, holes/tears caused by rodent damage were not associated with negative product perceptions [17]. Additional research may be needed to understand potential risks to ATSB acceptability when deployed under routine programmatic conditions. In the context of the Zambia trial, damaged ATSB stations were promptly removed and safely stored until incineration. If deployed under programmatic conditions, the type and frequency of damage observed in this trial may pose a threat to acceptability and/or create new concerns.

The ATSB station deployment and monitoring described here were part of a controlled cRCT aiming to achieve high coverage to assess potential product efficacy. It is not known what level of replacement would have been observed and coverage achieved under different installation and monitoring approaches, nor with different human resources and non-community-based monitoring models. When considering the costs of sourcing, transporting, storing, deploying, monitoring, and properly disposing of ATSB stations, early cost estimates suggest that ATSB stations in Zambia cost more to deploy than current standard vector control tools (Mancuso et al. in preparation). Further studies will be needed to document ATSB station replacement, coverage, and cost-effectiveness under different implementation and programmatic models.

The ATSB station replacement results reported here should be interpreted with consideration of minor limitations. While ATSB station damage was assessed by a trained and supervised community workforce, it is acknowledged that a degree of subjectivity was present in ATSB damage classification. ATSB monitors used their best judgement to classify ATSB damage according to project criteria and make replacement decisions. Detailed training materials, visual job aids, and photo reviews supported consistency in ATSB evaluation and data collection. Nevertheless, leaks, mold, bait depletion, and dirt were particularly challenging conditions to assess with objectivity given that some degree of bait leaking, mold growth, bait depletion, and dirt were allowable prior to meeting official criteria for replacement. If this ATSB Sarabi v1.2 product, or other ATSB stations, are deployed with replacement criteria due to damage, then clear and consistent guidelines, procedures, and resources will be required to operationalize routine and consistent practices.

## Conclusion

Although ATSBs may be a promising new paradigm for malaria vector control, maintaining high coverage of ATSB Sarabi v1.2 stations in good condition in a setting like western Zambia required intensive deployment and monitoring efforts. Optimizing the product to address threats to physical integrity, as well as implementation research to inform cost-effective deployment, optimal monitoring and replacements, and acceptance will be important to facilitate efficient scale-up of ATSB interventions.

## Data Availability

De-identified data are available from the corresponding author on reasonable request. Following publication of forthcoming secondary analyses of trial data, the deidentified trial dataset will be posted on a public repository.

## Abbreviations

ATSBs: Attractive targeted sugar bait stations
CI: Confidence interval
cRCT: Cluster randomized controlled trial
IRS: Indoor residual spraying
ITN: Insecticide treated net
NMEC: National Malaria Elimination Centre
WHO: World Health Organization
